# 3D printable tough hydrogel actuators

**DOI:** 10.1039/d6mh00947f

**Published:** 2026-07-28

**Authors:** Allison L. Chau, Esther Amstad

**Affiliations:** a Soft Materials Laboratory, Institute of Materials École Polytechnique Fédérale de Lausanne 1015 Lausanne Switzerland esther.amstad@epfl.ch

## Abstract

Nature autonomously actuates many of its structures in response to changes in environmental conditions. Inspired by nature, stimuli-responsive hydrogel-based actuators that wirelessly operate without external energy sources have been developed. By exploiting the tunable swelling behavior of hydrogels, these systems can actuate in the form of bending, twisting, folding, and even locomotion. However, for these materials to function effectively across a broad range of applications, their mechanical properties – especially their stiffness and toughness – must be improved to increase their actuation force and operational reliability. Addressing these mechanical performance challenges in hydrogel-based actuators would bring them closer to replicating the remarkable combination of mechanical toughness, resilience, and actuation observed in nature. This review outlines established toughening strategies for hydrogels and highlights advances in their additive manufacturing into actuators with well-defined structures and locally varying compositions. It concludes with a brief outlook on potential opportunities that arise if self-healing or improved fatigue resistance are incorporated into actuating systems.

## Abstract 2

Designing an untethered hydrogel actuator that simultaneously integrates high toughness, acceptable actuation, 3D printability, and biocompatibility remains a major challenge in the field of soft robotics.

## Graphical abstract

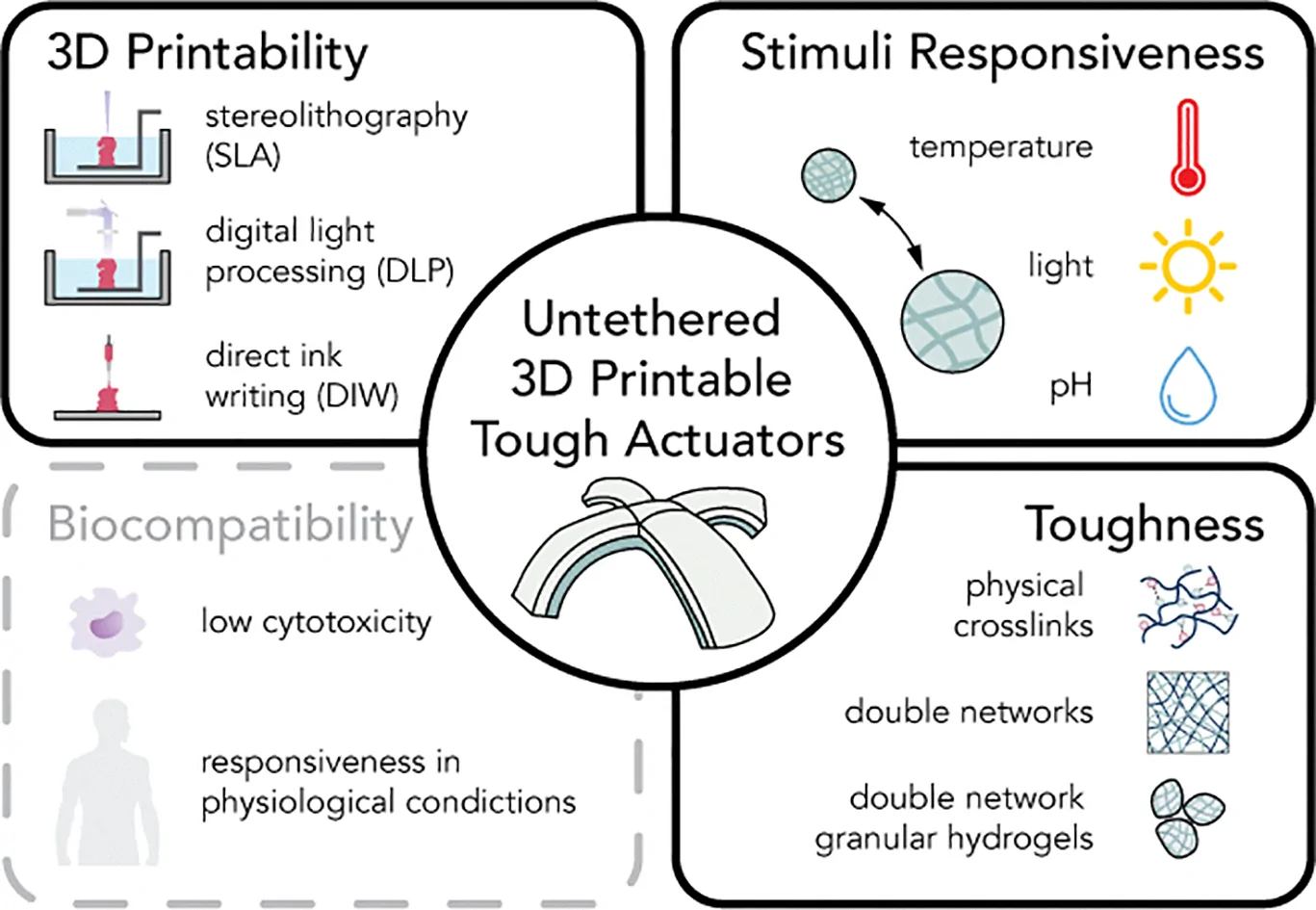

**Wider impact**

Many hydrogels respond to changes in their surroundings by altering their degree of swelling and hence, volume, which makes them promising materials for untethered actuators. However, currently available responsive hydrogels often exhibit poor mechanical properties, limiting their use in actuation. This review summarizes strategies to improve hydrogel mechanics through reinforcement with sacrificial or reversible bonds and discusses the metrics commonly used to quantify these properties. It further reviews additive manufacturing techniques frequently used to process hydrogels into actuators capable of well-defined motions and highlights how additive manufacturing can help accelerate response times. The review concludes that additively manufactured hydrogels are typically mechanically weak, whereas hydrogels with optimized mechanical properties are still primarily produced by casting. Finally, it outlines opportunities for additive manufacturing tough hydrogels for soft actuators with enhanced actuation force and durability.

## 1. Introduction

Soft robotics is a rapidly growing field, especially due to its potential in healthcare and food, as well as its capacity for biomimicry. The actuation mechanisms of soft robots are often inspired by the movements of plants,^1–5^ such as the bending of plant leaves driven by Turgor pressure^4,6–8^ or the snap-through behavior of Venus flytraps.^9–12^ In contrast to animals which often rely on muscles for movement, plant motion is driven by water transport^3^ and the ability of plants to change volume nonuniformly due to their heterogeneous, hierarchical structures.^2^

Pine cones are a prime example of water-induced actuation – seeds are encapsulated within pine cones in high humidity and released in response to decreased humidity. This hygroscopic behavior is attributed to the bilayer structure of the scales, where each layer has a heterogeneous distribution and alignment of cellulose microfibrils and porosity.^13–16^ This locally varying structure and composition creates differences in water uptake, generating a strain gradient that causes bending, allowing for release of the seeds. Other plants, including *Erodium cicutarium*,^17,18^*Pelargonium*,^19^ and wheat awn,^20^ utilize this hygroscopic mechanism to coil and uncoil their helical bilayer structure while drilling themselves into the soil. However, these actuation mechanisms can be quite slow, on the order of hours for pine cones,^14^ due to the dependence on water diffusion. Actuation time can be decreased if elastic instabilities are leveraged. The ultimate example of this mechanism is the Venus flytrap.^9,21–23^ When triggered by an insect, the Venus flytrap leaves will adapt from a convex curvature to a concave one within 100 ms,^9^ driven by Turgor pressure that leads to a heterogeneous strain distribution between the outer and inner epidermis.^21^

### 1.1. Soft robotic actuation

The elegant actuation mechanisms in plants have captured the imagination of researchers, who have replicated these behaviors in soft robots utilizing soft, flexible materials such as elastomers and hydrogels.^24–31^ Actuation is often achieved through pneumatic,^32–34^ hydraulic,^35–37^ or electrical^38–40^ power sources. While these systems can achieve fast actuation and high output forces, movement can be hindered due to wiring^41^ and failure can occur if the connections are not tightly sealed.^42^ To avoid this danger, untethered actuators, which rely on external stimuli to drive actuation, have been introduced.^41^

Hydrogels, which are polymeric networks swollen with water, are promising actuating materials because they undergo volumetric expansion due to osmotic pressure. Their water content, which scales with their degree of swelling, can be controlled by adjusting monomer type, polymer concentration, and crosslinker concentration. Additionally, many hydrogels are stimuli-responsive such that they increase or decrease their water content upon the application of an external stimulus – such as temperature,^43–47^ pH,^48–52^ light,^10,53–55^ or electricity^56,57^ – that changes the hydrophilicity of the polymer network. While this volumetric change can be exploited for linear actuation, it cannot bend. Bending actuation can be achieved if the responsive hydrogel is paired with a complementary soft material as a bilayer system. For example, stimuli-responsive hydrogels can be combined with passive non-stimuli-responsive hydrogels or elastomers.^58,59^

Poly(*N*-isopropylacrylamide) (pNIPAAm) is a widely used thermoresponsive^60^ hydrogel due to its reversible change in the degree of hydration at a lower critical solution temperature (LCST) around 32 °C.^61^ Once this LCST is surpassed at elevated temperatures, the hydrogen bonds between the polymer chains and water are disrupted and hydrophobic interactions between polymer chains dominate, leading to water expulsion.^62,63^ This feature was leveraged, for example, by covalently attaching pNIPAAm to poly(dimethylsiloxane) (PDMS) to form an actuator that could bend in two directions.^59^ Upon immersion in aqueous solutions, the actuator bent towards the PDMS layer as the pNIPAAm swelled while the hydrophobic PDMS did not. Once the temperature surpassed the LCST, bending reversed direction as pNIPAAm expelled water. The bending direction and degree were adjusted by tuning the thickness ratio of the layers and, to a lesser extent, the pNIPAAm polymer concentration.

Hydrogel actuation can be defined as any mechanical work or motion that occurs due to a stimulus, such as temperature, light, or pH. Depending on the application of the hydrogel actuator, they can be designed for movement or force generation. Many actuators are fabricated to undergo bending, twisting, or folding, or achieve locomotion such as crawling, swimming, or jumping. While these motions may not generate much usable force, they can be utilized in the design of soft grippers, smart textile, wearable devices, and sensors.^27^ Other actuators are designed to carry loads^64^ or produce high output forces,^8,42,65,66^ which can be useful in the design of artificial muscles or valves and pumps for microfluidic devices. For these applications, parameters such as work density^25^ and actuation stress (*i.e.*, blocking stress)^67^ are key. Work density is often quantified by measuring the displacement of a weight normalized by actuator volume or mass.^68,69^ Actuation stress is quantified as the force applied during actuation normalized by contact area.^70^ However, this review focuses on the bending capabilities of hydrogel-based actuators, as bending represents the fundamental deformation mode that governs more complex motions. Other aspects of actuation have been nicely summarized elsewhere.^30,71–73^

**Fig. 1:**
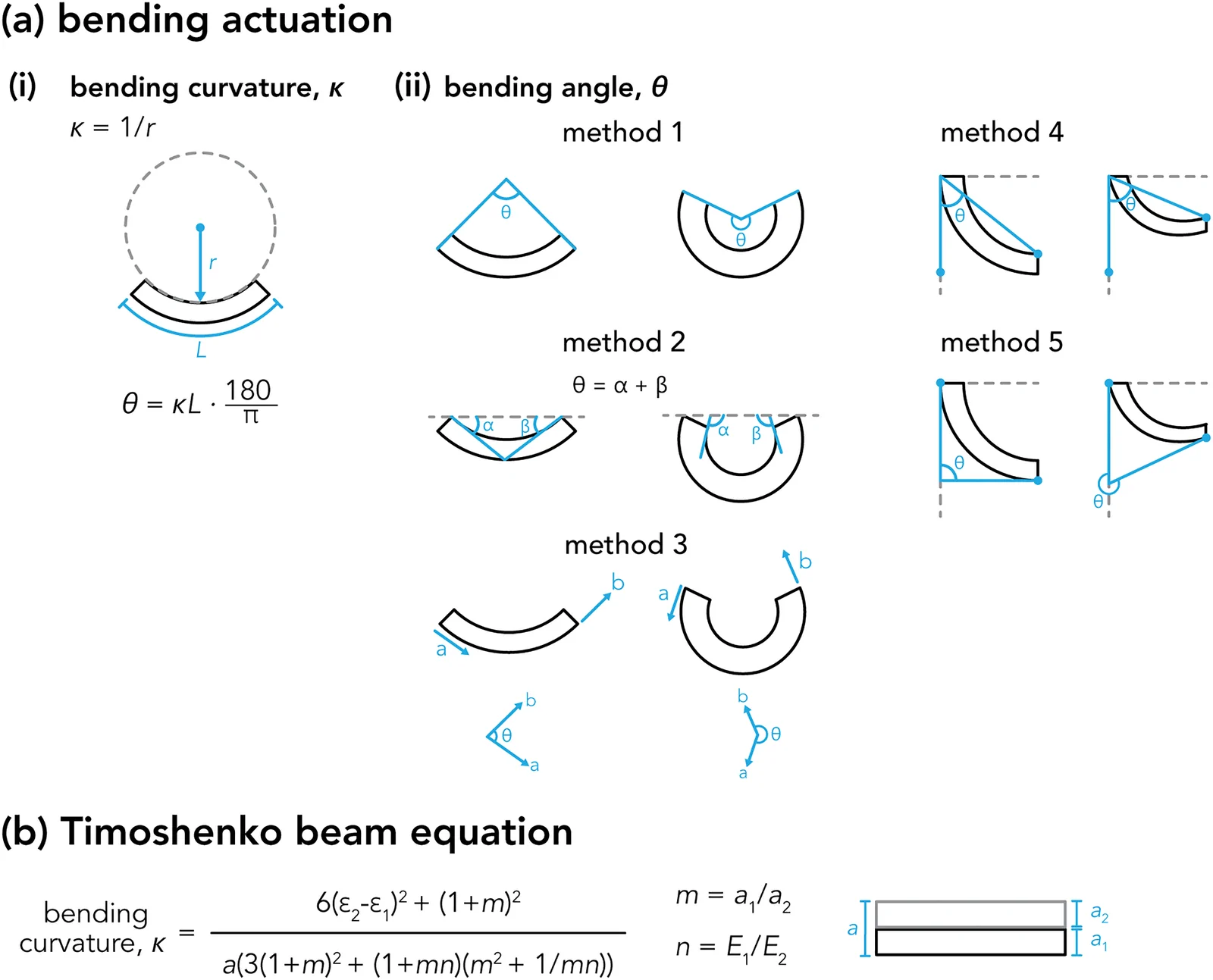
Schematics demonstrating the various methods utilized to estimate bending actuation. (a) Different techniques used to quantify bending actuation. (i) Experimental method to measure bending curvature (*κ*). If the length of the actuator (*L*) is known, the bending angle (*θ*) can be calculated using the stated equation. (ii) Experimental methods used to measure bending angle. Methods 1–3 (from ref. 75–82) produce similar results whereas Method 4 (from ref. 51 and 83–85) and Method 5 (from ref. 86) quantify bending angle using a fundamentally different approach as the actuators are fixed on one end, resulting in values that are not directly comparable. (b) The Timoshenko beam equation^88^ can predict bending curvatures for bilayer systems based on the elastic modulus (*E*), thickness (*a*), and strain (*ε*) of each layer.

The degree of bending can be quantified as bending curvature, *κ*, and can be measured with imaging software by fitting a circle around the curvature of the bent actuator. The inverse of the radius of the circle, *r*, is the bending curvature, *κ* = *r*^−1^.^74^ If the length of the actuator, *L*, is known, the bending angle, *θ*, can be determined. Bending angle can also be directly quantified by measuring the angle between two ends of an actuator beam, the extent to which the beam deviates from a straight line, or the angle between tangents at the two ends of the bent beam, as summarized in Fig. 1a. These methods, denoted as Methods 1–3 in Fig. 1a,^75–82^ will produce similar results. Other methods use beams that are fixed on one end only, such that their values cannot be directly compared, as exemplified with Methods 4^51,83–85^ and 5^86^ in Fig. 1a. Because of differences in the obtained absolute bending angles, it is critical to pay attention to the method used to calculate them. In yet some other cases, vertical or horizontal displacement of the actuator is used as a metric.^87^ Bending curvature can also be predicted for bilayer systems using the Timoshenko beam equation,^88^ as shown in Fig. 1b, which can be converted to bending angle. While experimental results do not perfectly align with those predicted by the formula due to the assumption that bending angle is independent of beam length,^89^ the equation provides useful guidelines for approximating bending angle based on the elastic modulus and thickness ratios of the two layers. Nevertheless, the diverse range of methods used to quantify actuation makes it challenging to compare results across literature.

**Fig. 2:**
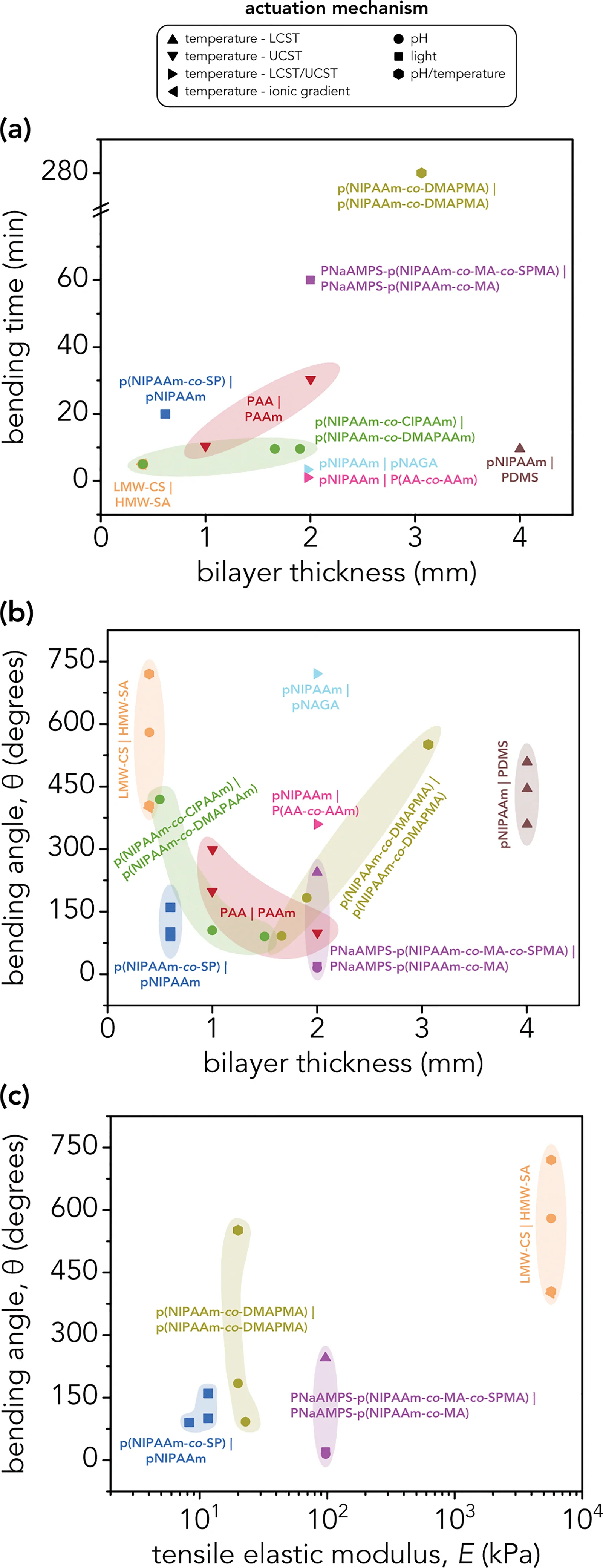
Ashby plots of bilayer hydrogel actuators comparing (a) bending time and (b) bending angle with bilayer thickness and (c) bending angle *versus* tensile elastic modulus grouped by bilayer composition. Most systems quantified bending angle utilizing Methods 1–3.^59,76,81,82,90,92^ However, the p(NIPAAm-*co*-DMAPMA) system (mustard circles and hexagon) was quantified with bending curvature, which was then converted to bending angle.^91^ Method 4 was used for the pNaAMPS-p(NIPAAm-*co*-MA-*co*-SPMA) system (purple diamond and triangle)^85^ and p(NIPAAm-*co*-CIPAAm) system (green circles),^51^ making it difficult to make a direct comparison with the rest of the data. There is no clear relationship between bending angle and bilayer thickness or tensile elastic modulus. Rather, the actuation behavior is primarily governed by the material system and actuation mechanism. Abbreviations: PAA|PAAm,^82^ LMW-CS|HMW-SA,^92^ pNIPAAm|PDMS,^59^ pNIPAAm|pNAGA,^81^ pNIPAAm|P(AA-*co*-AAm),^76^ p(NIPAAm-*co*-SP)|pNIPAAm,^90^ p(NIPAAm-*co*-DMAPMA)|p(NIPAAm-*co*-DMAPMA),^91^ p(NIPAAm-*co*-CIPAAm)|p(NIPAAm-*co*-DMAPAAm),^51^ pNaAMPS-p(NIPAAm-*co*-MA-*co*-SPMA)|pNaAMPS-p(NIPAAm-*co*-MA),^85^ PDMS – poly(dimethylsiloxane), PAA – polyacrylic acid, PAAm – polyacrylamide, pNAGA – poly(*N*-acryloyl glycinamide), P(AA-*co*-AAm) – poly(acrylic acid-*co*-acrylamide), LMW-CS – low molecular weight chitosan, HMW – SA: high molecular weight sodium alginate, PNaAMPS – poly(2-acrylamide-2-methyl propane sulfonate acid sodium), pNIPAAm – poly(*N*-isopropyl acrylamide), p(NIPAAm-*co*-SP) – poly(*N*-isopropyl acrylamide-*co*-spiropyran), p(NIPAAm-*co*-MA) – poly(acrylic acid-*co*-methyl acrylate), p(NIPAAm-*co*-MA-*co*-SPMA) – poly(acrylic acid-*co*-methyl acrylate-*co*-spiropyran), p(NIPAAm-*co*-CIPAAm) – poly(*N*-isopropyl acrylamide-*co*-2-carboxyisopropylacrylamide), p(NIPAAm-*co*-DMAPMA) – poly(*N*-isopropyl acrylamide-*co-N*-[3-(dimethylamino) propyl] methacrylamide), p(NIPAAm-*co*-DMAPAAm) – poly(*N*-isopropyl acrylamide-*co-N,N’*-(dimethylamino) propyl acrylamide).

The bending angle of bilayer actuators can range from 20 to 720° with actuation times varying from 1 to 280 min (Fig. 2a).^51,76,81,82,85,90–92^ For example, the bending angle of a hydrogel bilayer was increased by combining a pNIPAAm layer, which exhibits an LCST, with a poly(acrylic acid-*co*-acrylamide) (P(AA-*co*-AAm)) layer with an upper critical solution temperature (UCST).^76^ In contrast to LCST systems, hydrogels with a UCST uptake water at elevated temperatures due to the disruption of hydrogen bonds between the polymer chains, enabling the network to swell.^93^ Similar to the actuation of mimosa leaves, water was internally exchanged between layers as the LCST hydrogel expelled water that was subsequently absorbed by the UCST hydrogel. This large swelling difference induced actuation, allowing the system to bend 360° within minutes, as shown by the pink triangle in Fig. 2b. Similarly, the bending angle of bilayers composed of poly(*N*-isopropylacrylamide-*co-N*-[3-(dimethylamino) propyl] methacrylamide) (p(NIPAAm-*co*-DMAPMA)), with varying DMAPMA concentration in each layer, was increased by simultaneously adjusting pH and temperature to maximize the swelling difference between the two layers, as shown by the mustard hexagon in Fig. 2b. The bending angle of a bilayer composed of a low molecular weight chitosan (LMW-CS) layer and a high molecular weight sodium alginate layer (HMW-SA) was increased by introducing two stimuli simultaneously (orange shapes).

Bending relies on the differences in swelling degree of the two hydrogel layers, which strongly depend on their composition. Hence, no clear relationship between bending angle and bilayer thickness or tensile elastic modulus can be seen, as shown in Fig. 2. Rather, bending actuation depends on the strain difference between the hydrogel layers, which is influenced by swelling, and generally increases with increasing strain difference, as demonstrated by the Timoshenko beam equation.

Reliable and repeatable bending of bilayer structures requires strong interlayer adhesion, which is influenced by the bilayer fabrication process and interlayer composition. Bilayer structures are commonly fabricated by casting the first layer, which is partially or fully cured. The precursor solution of the second layer is then added on top, allowing it to infiltrate the first layer, thereby generating an interpenetrating polymer network that ensures strong adhesion between the two layers.^76,81,82,84,85,90^ Alternatively, bilayers can be attached through electrophoresis, which forms polyion complexes at the interface,^51,94^ or with adhesives.^14,56^ However, the interfacial adhesion achieved through these processes is often limited, risking delamination. Additionally, mismatches in mechanical properties between adjacent layers tend to induce stress concentrations at the interface, which often accelerate delamination.

Casting is cost efficient and offers good control over the overall composition of materials. However, casting is limited to fabricating simple geometries and offers little control over the local composition and structure. Nature produces materials with hierarchical and gradient structures that are well-defined over many orders of magnitude and, therefore, often have excellent mechanical properties. To more closely mimic the performance of natural materials, nonresponsive and stimuli-responsive hydrogels have been 3D printed into actuators, as has nicely been reviewed.^43,95–100^ These actuators achieve bending through bilayer structures composed of an active and passive layer,^79,101,102^ by leveraging gradients in crosslinking density^103–105^ or through anisotropic swelling^78^ and alignment of fibers.^106^ Because these actuators were printed into complex structures, they were able to achieve not only bending but also more complex actuation modes than cast structures, including uniaxial elongation or radial expansion.^75,107,108^

### 1.2. Limitations of untethered soft actuators

Untethered soft actuators typically are slow because they rely on the diffusion of water. Actuation can be accelerated by reducing the thickness of the actuator, as demonstrated in Fig. 2a, generating connected micrometer-sized pores through porogens^101,109^ or phase separation,^83^ or 3D printing macroscopic porous lattice structures.^86,108^ However all of these methods weaken the mechanical properties of the system, which limits actuation force. Due to the low elastic modulus of single network hydrogels, which is on the order of a few to 100s of kilopascals, the swelling and contraction forces for most hydrogel actuators are less than 1 N,^8,42^ even though the osmotic pressure that drives swelling can reach megapascals.^110^ In comparison, the contractile actuation force generated by Torrey pine cone scales bending is an order of magnitude higher at ∼10 N.^14^

**Fig. 3:**
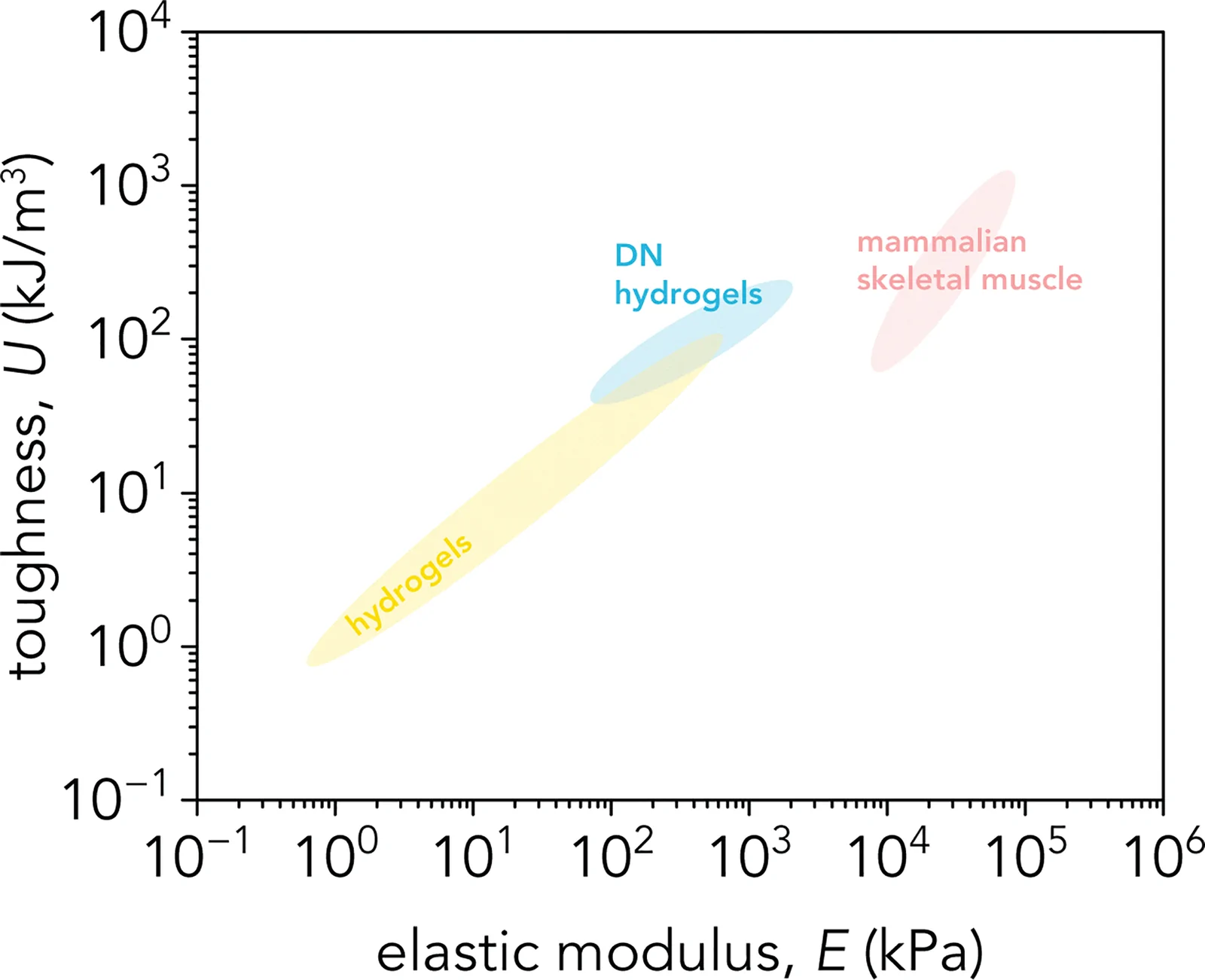
Ashby plot comparing the elastic modulus and toughness of single network hydrogel actuators, tough double network (DN) hydrogels, and mammalian skeletal muscles. Values taken from ref. 65,66 and 115–119.

Actuation force increases with increasing hydrogel stiffness.^111^ Yet, in hydrogels, an increase in stiffness is often paired with a reduction in toughness,^112^ strain at break,^113,114^ and fatigue resistance.^113^ While the reduced toughness may trigger catastrophic failure during operation, the reduced strain at break limits its extensibility and the decreased fatigue resistance reduces the lifetime of these materials. As a result of this trade-off between different mechanical properties, untethered soft actuators typically exhibit stiffnesses that are far below that of mammalian skeletal muscles, as summarized in Table 1 and Fig. 3.

**Table 1:** Comparison of the elastic modulus and toughness of single network hydrogel actuators, tough double network (DN) hydrogels, and mammalian skeletal muscles. Values taken from ref. 65,66 and 115–119

	Typical hydrogel actuators^113,118^	Tough double network (DN) hydrogels^113^	Mammalian skeletal muscles^65,66,116,119^
Elastic modulus	<MPa	0.1–1 MPa	10–60 MPa
Tensile strength	<MPa	1–10 MPa	—
Strain at break	<100%	1000–2000%	—
Toughness	∼1–10 kJ m^−3^	∼100 kJ m^−3^	∼100–800 kJ m^−3^
Fracture toughness (*i.e.* fracture energy)	0.1–10 J m^−2^	100–4400 J m^−2^	2490 J m^−2^
Actuation stress (*i.e.* blocking stress)	2 kPa	—	100–350 kPa

For actuators to reliably operate, they must absorb energy without catastrophically failing. The amount of energy a material can absorb before it fails can be quantified by toughness, which is most frequently quantified as the area under a tensile stress–strain curve for hydrogels.^114^ However, the toughness of typical single network hydrogels is an order of magnitude below that of mammalian skeletal muscles, as shown in Fig. 3. Toughness can be increased by introducing reversible or sacrificial bonds, as has been nicely shown, for example, in double network hydrogels.^118,120–122^ However, many of these toughening mechanisms limit the actuation range of the material because they constrain volumetric expansion and contraction or utilize stimuli-responsive sites.^1,67,86^ Consequently, designing a hydrogel that simultaneously achieves high toughness, acceptable actuation, and 3D printability is challenging. The remainder of this review will focus on the advances of 3D printing tough hydrogel actuators. First, the most frequently utilized 3D printing methods will be discussed along with their advantages and limitations. Then recent advances in the design of 3D printed tough hydrogel actuators will be considered. The review ends by mentioning some challenges and providing a short outlook for the field.

## 2. 3D printing methods for hydrogel actuators

Additive manufacturing has the potential to build hydrogels that combine stiffness, toughness, and fatigue resistance within a single system by locally varying composition to integrate hydrogels whose mechanical properties have been optimized for different parameters. Hydrogels can be 3D printed through inkjet, extrusion, and light-based printing as has been nicely summarized.^123–130^ Yet, hydrogel actuators are most commonly 3D printed through vat photopolymerization, where vat refers to the reservoir containing photocurable precursors, and extrusion printing due the simplicity, versatility, and accessibility of these techniques, as summarized in Table 2.

**Table 2:** Comparison of vat photopolymerization and extrusion printing techniques for 3D printing hydrogel actuators

	Technique	Ink requirements	Resolution	Advantages	Disadvantages
Vat photopolymerization	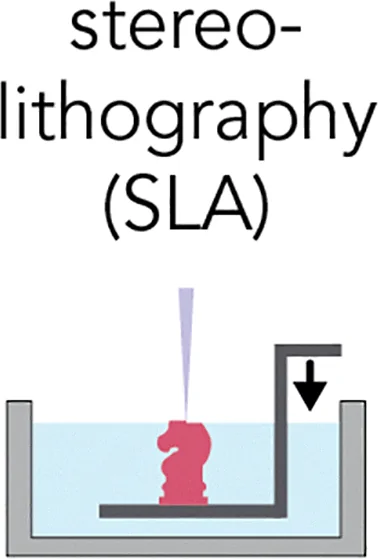	• Photocurable resin^124^	• Lateral: 5–50 µm, dependent on laser spot size^120,125^	• High resolution – enables smaller feature sizes	• Anisotropy may occur within layers due to curing gradient^120^
		• Viscosity: <4 Pa s^120^	• Vertical: dependent on light penetration depth^125^	• Smooth surface finish	• Difficult to print multiple inks^120,124^
			• Fabrication rate: ∼10^5^ mm^3^ h^−1 ^^128^		• Additives may increase viscosity
			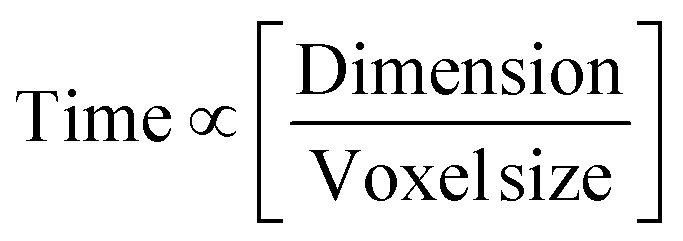		• Large amounts of precursor solution needed (∼500 mL) and most remains unused^129^
	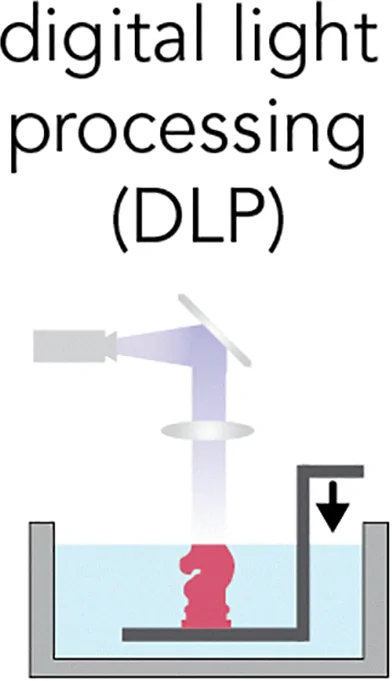	• Photocurable resin^124^	• Lateral: 5–50 µm, dependent on projector pixel size^120,125,130^	• Faster printing speed than SLA^120,125^	• Lower resolution compared to SLA
		• Viscosity: <4 Pa s^120^	• Vertical: dependent on light penetration depth^125^	• Typically less affected by oxygen inhibition than SLA^125^	• Difficult to print multiple inks^120,124^
			• Fabrication Rate: ∼10^6^ mm^3^ h^−1 ^^128^		• Additives may increase viscosity
			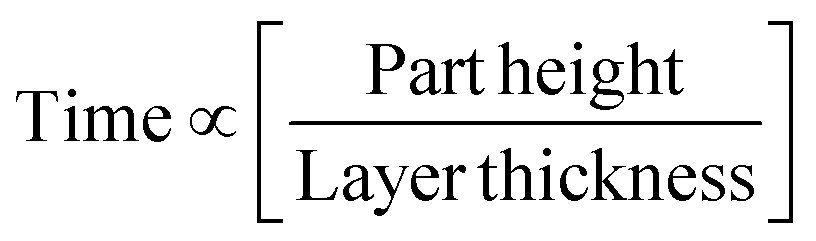		• Large amounts of precursor solution needed (∼500 mL) and most remains unused^129^
Extrusion 3D printing	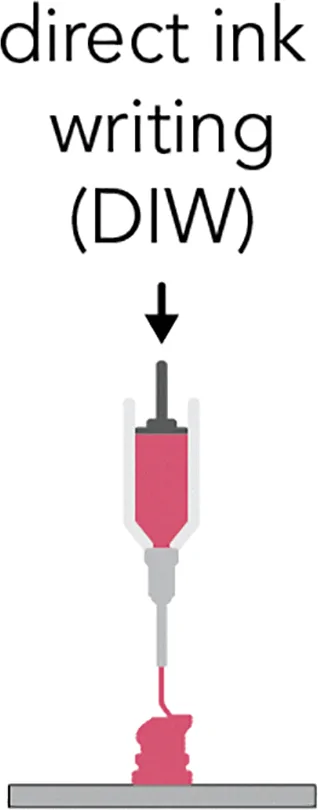	• Viscoelastic	• Lateral: 1 – 150 µm, dependent on nozzle diameter and printing speed^93,120,124^	• Material versatility^124^	• Slower printing speeds and lower resolution compared to SLA and DLP
		• Shear thinning^122^	• Vertical: dependent on ink yield stress and capillary forces^131^	• Easy to co-print multiple inks	• Bubbles trapped between layers^122^
		• Viscosity: 0.1–1000 Pa s^96^	• Fabrication Rate: ∼10^3^–10^4^ mm^3^ h^−1 ^^132^	• Anisotropic alignment of fillers	• Difficult to print overhangs^122^
		• Storage modulus (*G*′): 10^4^–10^5^ Pa^122^	• Time ∝ (print speed) × (nozzle diameter)^2^	• Requires less ink than SLA and DLP^129^	• Unpolymerized structures may collapse under the weight of subsequent layers^124^
		• Yield point (*G*′ = *G*″): 500–2500 Pa^122^			• Nozzle clogging due to viscous inks or fillers^133^
		• Shape fidelity: dependent on tan *δ* (*G*′ > 1)^93^			

### 2.1. Vat photopolymerization

**Fig. 4:**
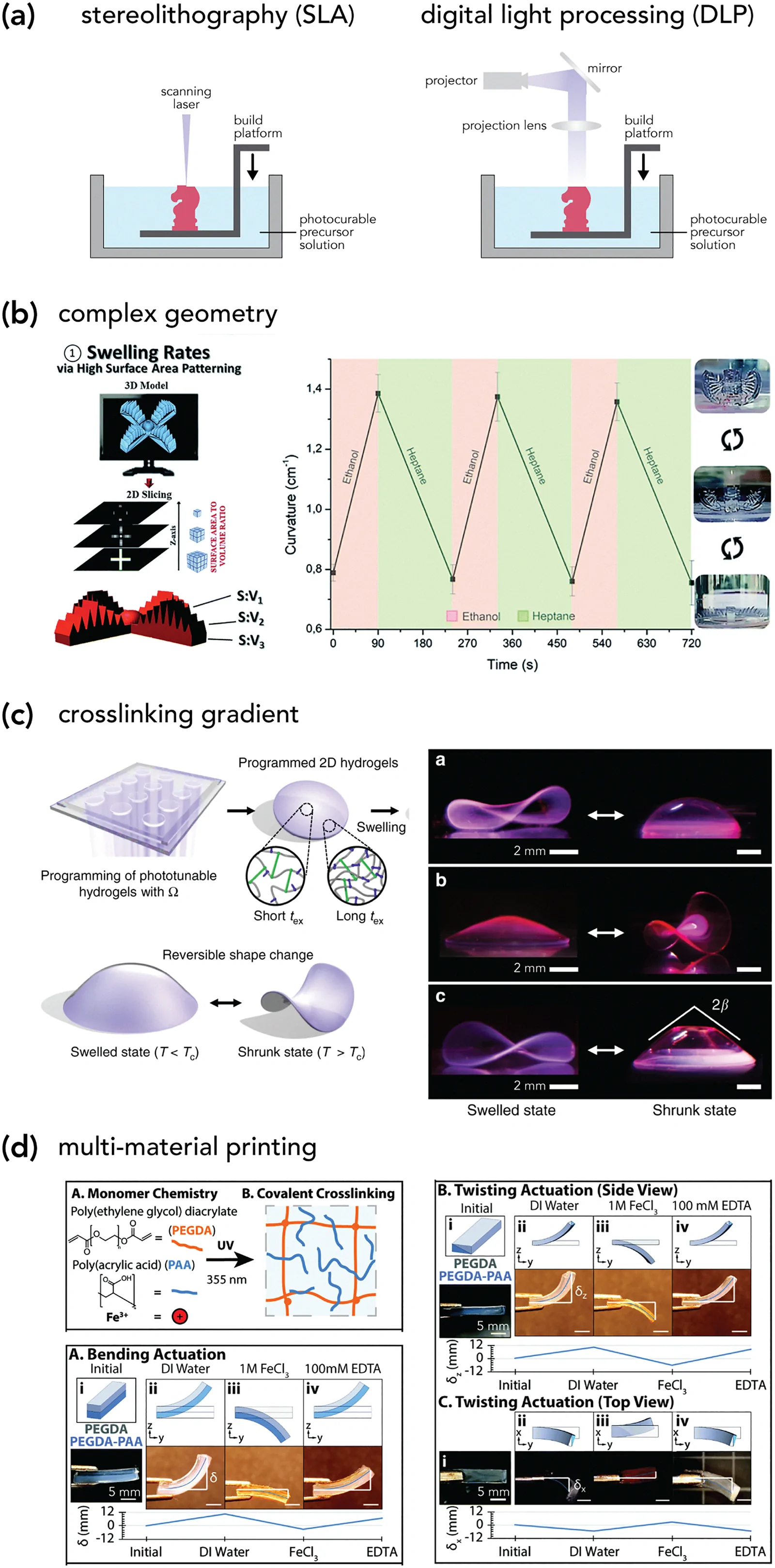
Demonstration of vat photopolymerized 3D printed hydrogel actuators. (a) Schematics of stereolithography (SLA) and digital light processing (DLP) 3D printing. (b) SLA-printed hydrogel actuator with a surface area-to-volume ratio gradient through the sample thickness demonstrating the complex geometry that is achievable through vat photopolymerization. Adapted and used with permission of Royal Society of Chemistry from ref. 74; permission conveyed through Copyright Clearance Center, Inc. (c) Example of a hydrogel actuator DLP-printed from a single ink. By varying photopolymerization times, crosslink density gradients were introduced into the hydrogel to achieve specific bending patterns. Scale bars: 2 mm. Adapted with permission from ref. 104. Copyright 2018 Springer Nature. (d) Hydrogel actuator printed *via* SLA demonstrating multi-material printing. By changing the bilayer geometry, different actuation modes were achieved. Scale bars: 5 mm. Adapted and used with permission of Royal Society of Chemistry from ref. 87; permission conveyed through Copyright Clearance Center, Inc.

Vat photopolymerization is a popular 3D printing technique for hydrogels due to its scalability, fast fabrication, and high printing resolution.^131^ Two of the most common vat photopolymerization techniques used to fabricate hydrogel actuators are stereolithography (SLA) and digital light processing (DLP). Both processes are based on a bath of low viscosity (<4 Pa s)^123^ photocurable hydrogel precursor solution that is locally polymerized with a specific light wavelength (*e.g.*, UV). The key difference in these 3D printing techniques is the light source – SLA cures the resin point by point with a laser whereas DLP employs a projected light image to cure the resin layer by layer, making DLP the faster printing method, as shown in Fig. 4a. In both cases, the nominal lateral printing resolution ranges from 5–50 µm. In SLA, the printing resolution is limited by the laser spot size whereas in DLP, it is controlled by the projector pixel size. The vertical resolution is further influenced by the *z*-axis stage of the printer as well as curing penetration depth, which is dependent on the light absorption and curing characteristics of the precursor solution^128^ and can be estimated with the Beer–Lambert equation.^132^ The choice between SLA and DLP depends on the trade-off between print quality and speed: SLA enables smaller feature sizes and higher feature fidelity but is generally slower than DLP.^127^

SLA and DLP require low viscosity liquid precursor solution to easily spread and replenish the build layer.^127^ Most acrylated and methacrylated monomers such as acrylic acid and poly(ethylene glycol) dimethacrylate (PEGDMA) fulfill these viscosity requirements without further modification, as has been nicely summarized.^123,126^ To prevent premature polymerization of the curing bath and control the laser penetration depth, radical inhibitors such as hydroquinone^74^ and light absorbers such as Sudan I dye^133^ are often added to the resin to improve vertical resolution by reducing light scattering.^97,123,127^ To achieve optimal mechanical properties of the printed parts, residual resin is washed off and the sample is post cured with UV light to polymerize any leftover unreacted monomers.^126^

3D printing allows for more complex geometries than would be possible *via* casting. For example, a multi-arm gripper composed of NIPAAm was SLA-printed with a raised ridge pattern across the length of each arm, and the surface area-to-volume (S : V) ratio was controlled in step-like patterns throughout the z-axis, as shown in Fig. 4b.^74^ Since the swelling rate is diffusion dependent and hence, linearly scales with the surface area-to-volume ratio, this parameter was controllably varied across the thickness of the gripper to impose a swelling gradient. When placed in ethanol, the arms curved upwards due to greater swelling in the high surface area features. Once placed in heptane, the arms reversed and attained its initial flat geometry.

Hydrogel actuators can be printed from a single ink. This can be achieved by introducing crosslink density gradients during the printing process by modulating light exposure time or intensity within or between print layers. For example, poly(hydroxyethyl acrylate) (pHEA) and poly(hydroxyethyl methacrylate) (pHEMA) structures were generated with DLP by modulating the light exposure time at each pixel level.^103^ By varying polymerization time, the crosslink density, and hence stiffness, was spatially controlled. Similarly, pNIPAAm structures incorporating two different crosslinkers were DLP-printed.^104^ Differences in crosslinker length and reaction kinetics created compositional gradients within the material, which led to controlled buckling and shape changes due to anisotropic swelling when immersed in water, as demonstrated in Fig. 4c. Yet, the complexity of these actuators and their actuation is limited by the narrow range of stiffness gradients that can be achieved if only one ink is used.

Larger gradients in stiffness can be achieved if actuators are made from multiple materials. Such actuators can be processed through vat photopolymerization^134,135^ by exchanging the resin bath between the photopolymerization of adjacent layers manually^36,132,136^ or autonomously.^123,132,136^ In both cases, this exchange is time consuming and often requires a cleaning step to remove excess uncured ink to prevent cross-contamination.^136^ Yet, this resin exchange enables the formulation of bilayer actuators. For example, bilayers composed of a poly(ethylene diacrylate) (PEGDA) layer connected to a double network layer of poly(ethylene diacrylate)-poly(acrylic acid) (PEGDA-PAA) were 3D printed *via* SLA. When printed as two parallel horizontal slabs, the bilayer bent in one direction when immersed in water due to anisotropic swelling. Once water was exchanged for an aqueous FeCl_3_ solution, the bilayer bent in the opposite direction due to ionic crosslinking between the Fe^3+^ ions and carboxylic groups in the PAA. By contrast, when printed as two triangular wedges conformally interlocked diagonally across the axial cross-section, the structure twisted in multiple directions upon submersion in water and aqueous FeCl_3_ solution, as shown in Fig. 4d.^87^

Multi-material SLA and DLP printing typically involves controlled compositional changes between successive layers, while the composition within a single layer remains uniform. In-plane compositional variation can be achieved if the vat baths are exchanged multiple times during the printing of a single layer. Algorithms have been developed to optimize the number of vat changes, but care must be taken to prevent misalignment between inks and achieve good polymerization across the multiple ink interfaces. Additionally, laser shadowing can occur, where the polymerizing laser light is obstructed by a previously printed section.^137,138^

In summary, vat photopolymerization is a versatile, fast 3D printing technique that works well with most photopolymerizable hydrogel precursor solutions since there are very few rheological requirements. It is a well-suited technique to fabricate actuators requiring crosslink density gradients, simple bilayer structures, a good surface finish, overhanging parts, or sharp, high surface area regions. However, difficulties can arise if more complex multi-material patterning is required or if anisotropic alignment of fillers is desired. Additionally, actuators might suffer from poor adhesion between layers due to uneven or partial curing. Some of these limitations can be addressed if actuators are fabricated through direct ink writing, albeit at the expense of printing resolution and speed.

### 2.2. Direct ink writing (DIW)

**Fig. 5:**
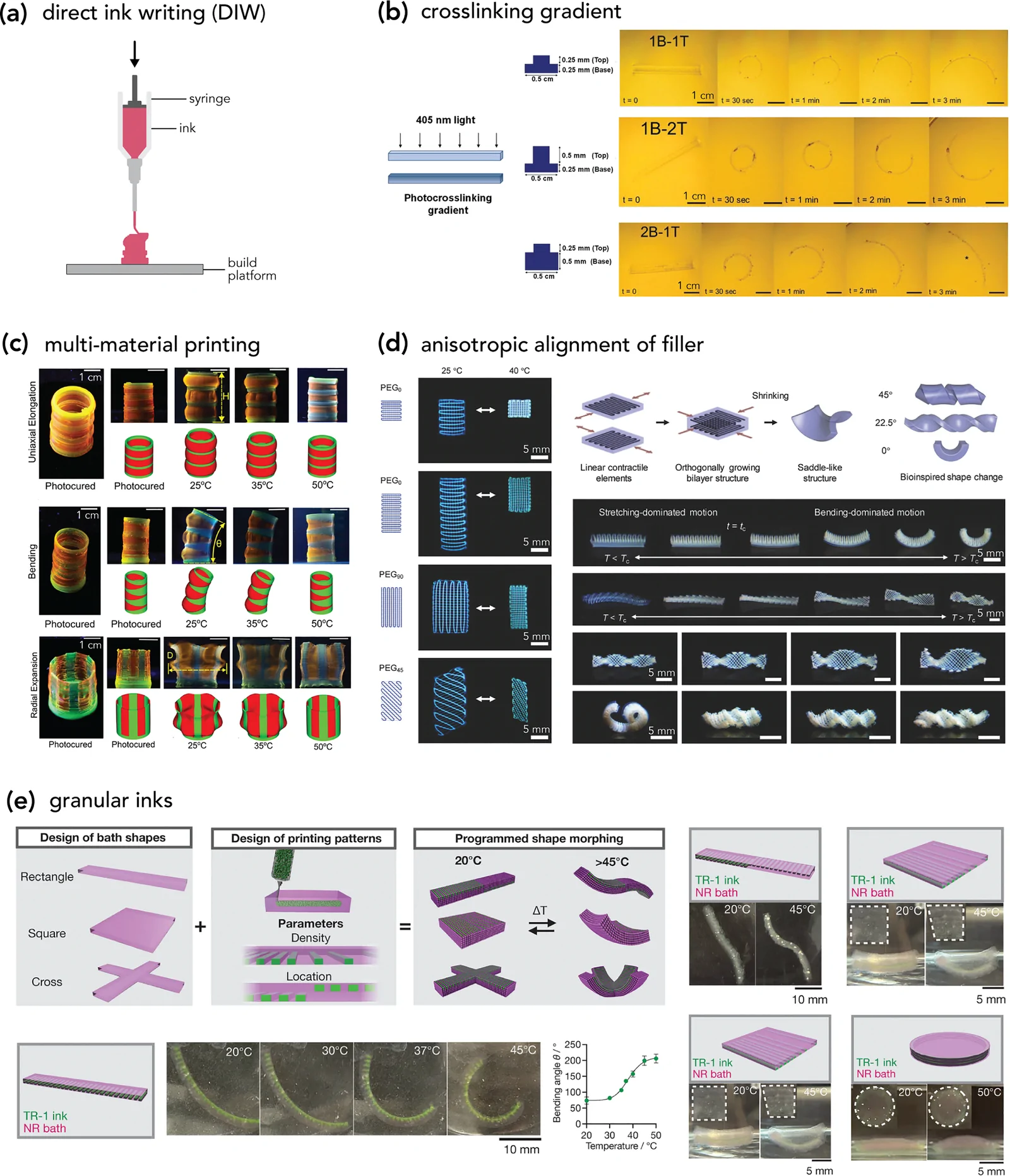
Demonstration of direct ink written 3D printed hydrogel actuators. (a) Schematic of direct ink writing (DIW). (b) Example of a DIW-printed actuator with crosslinking gradient. Scale bars: 1 cm. Adapted with permission from ref. 78. Copyright 2023 John Wiley & Sons. (c) DIW-printed pNIPAAm-PAAm hydrogel actuators demonstrating multi-material printing. Scale bars: 1 cm. Adapted with permission from ref. 107. Copyright 2019 American Chemical Society. (d) Poly(ethylene glycol diacrylate) (PEGDA) patterned in a pNIPAAm matrix. Due to the anisotropic placement of the nonresponsive PEGDA, shape morphing occurred upon elevated temperatures. Scale bars: 5 mm. Adapted from ref. 108 Copyright 2018 John Wiley & Sons. (e) DIW printing of granular inks to form a bilayer actuator. Scale bars: 5 or 10 mm. Adapted with permission from ref. 75. Copyright 2024 John Wiley & Sons.

Direct ink writing (DIW) is an extrusion-based 3D printing technique where hydrogel precursor solution is extruded from a nozzle to form structures layer-by-layer that are photopolymerized after each layer or in bulk, as demonstrated in Fig. 5a. The storage modulus of the ink should be around 10^4^–10^5^ Pa^125^ while the viscosity can range between 0.1–1000 Pa s.^100^ Hydrogel precursors can be direct ink written into aqueous solutions or air if they are shear thinning, exhibit low yield stress (500–2500 Pa), and have fast stress recovery.^125^ To satisfy these rheological requirements, rheological modifiers, such as LAPONITE®, a synthetic layered silicate nanoclay,^102,106,107^ and methyl cellulose,^78,139^ are often added to the precursor solution. Yet, printed structures can collapse before polymerization due to the weight of the subsequent layers, limiting the maximum print height.^127^ Furthermore, it is difficult to print structures with overhangs.^125^ These limitations can be overcome if the ink is printed into a support bath designed to retain the shape of the printed structures.^140^ However, the support bath has its own rheological requirements that need to be fulfilled. It must be shear thinning, and the shear elastic modulus and yield stress of the support bath must be an order of magnitude lower than those properties of the ink.^141^ The lateral resolution of direct ink writing depends on nozzle diameter and printing speed and typically ranges from 1–150 µm,^97,123,127^ while the vertical resolution is dependent on the ink yield stress and capillary forces.^142^

Controlled motion of direct ink written hydrogels can be achieved by introducing crosslinking gradients^74^ by varying light exposure time or intensity across the thickness,^78^ as shown in Fig. 5b. For example, the exposure of only the top surface of an uncrosslinked DIW-printed structure resulted in a crosslinking gradient through the thickness of the structure due to the reduction in light intensity with increasing light penetration depth.^78^ Moreover, inks can be more readily changed *in situ* if printed through DIW compared to SLA and DLP as multiple inks can be co-printed between consecutively deposited layers^43,101,102^ or within the printing plane.^75,143^ For instance, mixed ink actuators composed of temperature-responsive pNIPAAm and nonresponsive poly(acrylamide) (PAAm) were DIW-printed in patterns that would be difficult and time-consuming to access with vat polymerization, as exemplified in Fig. 5c.^107^ Multi-ink printing within a plane was also demonstrated through the formation of a bilayer consisting of an active pNIPAAm layer and mixed-ink passive PAAm layer.^143^ The passive layer was composed of two PAAm inks with different crosslinker concentrations. This multi-ink strategy enabled spatial tuning of the passive layer stiffness, thereby directing bending actuation.

Direct ink writing is also well suited to introduce anisotropy into structures. Hydrogels are often reinforced with nanoparticles and fibers to enhance their mechanical properties.^106,144–147^ If the fillers are anisotropic, such as high aspect ratio fibers^106^ or graphene oxide,^148^ they can be oriented along the printing direction through shear-induced alignment as ink is extruded through the nozzle during the DIW process. This anisotropic alignment of fillers often leads to actuation or shape morphing without the need for bilayer structures, as swelling is constrained in one axis due to increased stiffness, thereby provoking anisotropic volume change.^149^ For example, stiff cellulose nanofibrils were aligned within a soft PAAm matrix, resulting in a two-fold increase in stiffness and four-fold decrease in swelling in the longitudinal direction compared to the transverse direction.^106^ This swelling anisotropy caused the printed structures to curl and bend upon immersion in water. Similarly, passive hydrogels can be patterned within a stimuli-responsive hydrogel matrix to achieve actuation. For instance, unresponsive poly(ethylene glycol diacrylate) (PEGDA) was patterned in a pNIPAAm matrix to induce bending and twisting at elevated temperatures due to anisotropic shrinking of the pNIPAAm matrix around the PEGDA patterns, as demonstrated in Fig. 5d.^108^

Direct ink writing allows for a broader range of ink viscosities compared to SLA and DLP. This feature has been leveraged to 3D print granular hydrogels composed of densely packed hydrogel-based microparticles, commonly referred to as microgels.^150–153^ The printed granular structures can then be solidified by connecting adjacent microgels at their surfaces^75^ or through a second network that interpenetrates and covalently connects them.^83^ Hydrogel-based granular bilayer actuators have been produced by direct ink writing a thermoresponsive granular ink onto a passive granular ink and subsequently covalently bonding all microgels at their surfaces, as shown in Fig. 5e.^75^ By tuning the density and vertical position of the thermoresponsive hydrogel within the actuator, the bending angle was tuned from 25° to 125°. To increase complexity, a third responsive ink was added to the actuator to achieve stepwise bending at varying temperatures. Note that in this case, the printing resolution is limited by the size of the microgels as the nozzle diameter must be 10–100 times greater than the microgel size.^75,100^

In conclusion, DIW is better suited than SLA and DLP for fabricating actuators with complex shapes and spatially varying composition, as well as those requiring anisotropic filler alignment. In contrast, SLA and DLP are more advantageous for fabricating actuators with crosslinking gradients or bilayer structures due to faster fabrication speeds. Yet, many of the hydrogel precursor solutions used during vat photopolymerization are composed of low molecular weight monomer solutions that form brittle networks when polymerized.^154^ Indeed, most of the hydrogel actuators discussed in this section were optimized for actuation, often at the expense of their mechanical properties. The following section will discuss common toughening mechanisms and the recent advancements in the design of tough hydrogel actuators.

## 3. 3D printed tough hydrogel actuators

**Fig. 6:**
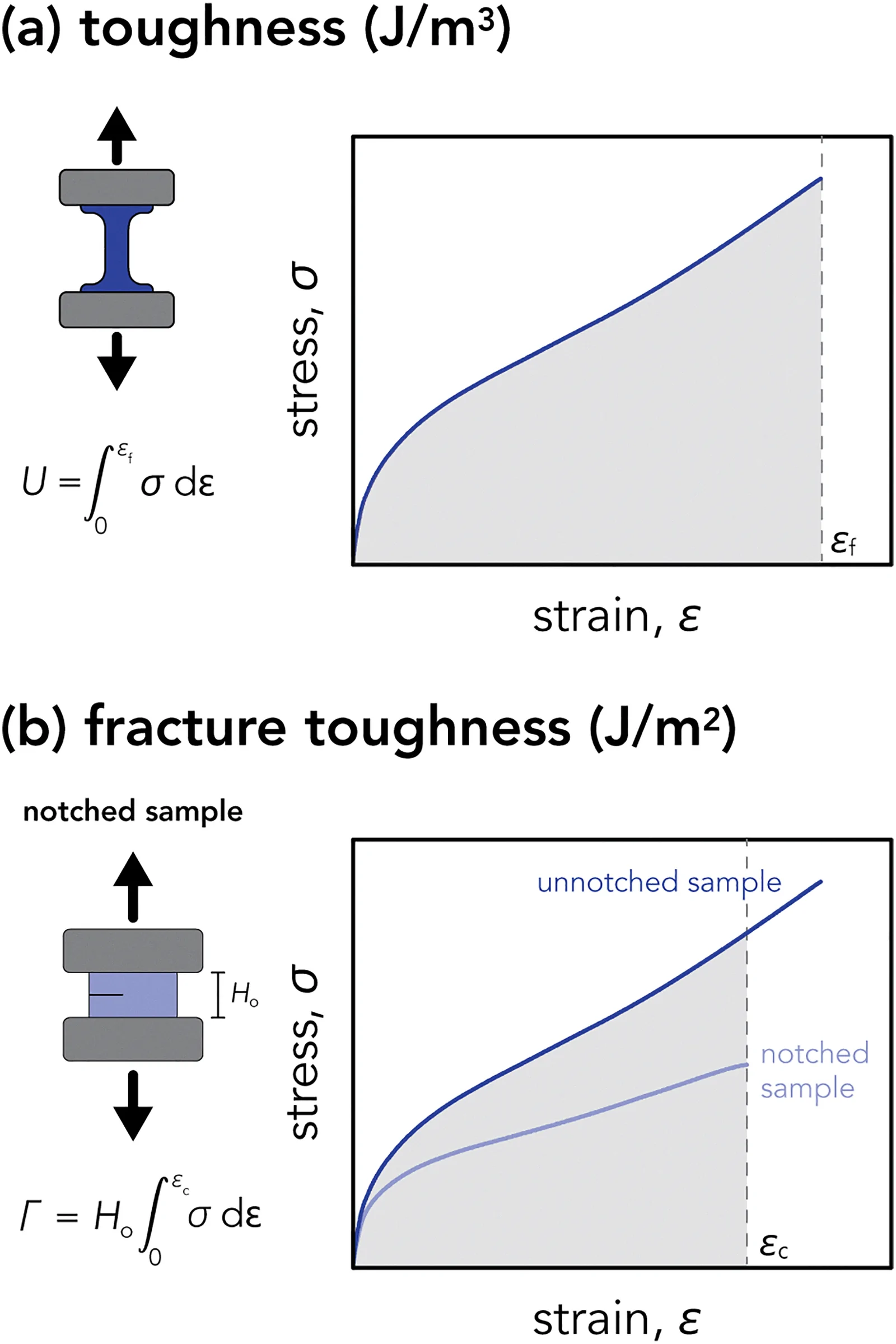
Schematics demonstrating two different methods for quantifying toughness in hydrogels. (a) Toughness, also known as strain energy density, can be quantified as the area under the stress–strain curve and is the amount of strain energy per unit volume that a material can absorb before fracture. (b) Fracture toughness is the amount of energy that is required to propagate a crack.

Toughness is the ability of a material to absorb energy without failure. In hydrogels, toughness can be defined as the energy stored within a material before failure per unit volume and can be quantified as the area under a tensile stress–strain curve, as shown in Fig. 6a. Toughness can also be defined as fracture toughness, which is the resistance of a material to crack propagation. In classic fracture mechanics, fracture toughness refers to *K*_c_, the stress intensity factor, which is valid for linear elastic materials such as metals and ceramics. However, classic fracture mechanics cannot be directly applied to viscoelastic polymers. This limitation was partially addressed by introducing an energy-based approach to the fracture mechanics of rubber through the fracture energy, which is the amount of energy needed to generate new fracture surfaces required to propagate a crack. Through this approach, it was demonstrated that a crack will grow once enough energy is available to create a new surface rather than when a critical stress value is surpassed.^155^ Although initially developed for rubber, this energy-based framework has since been extended and widely applied to hydrogels.^117,156^

The fracture energy of hydrogels can be quantified using pure shear tests, in which notched and unnotched samples are subjected to tensile loading until failure. The failure strain of the notched sample defines the integration limit used to calculate the fracture toughness of the unnotched sample, as shown in Fig. 6b. The toughness of most hydrogel actuators is reported as the area under the stress–strain curve, with very few studies providing fracture toughness values. Therefore, we compare hydrogel actuator toughness measured as the area under the stress–strain curve.

### 3.1. Common toughening mechanisms

The key limitations of many 3D printed hydrogel actuators are their poor mechanical properties: layer-by-layer deposition typically produces materials that are weaker than bulk-processed counterparts of identical composition, such as those made by casting.^157^ This weakness arises mainly from poor interlayer adhesion^158^ and restricted material options. This limitation can be addressed by designing tough hydrogels. Toughening mechanisms of bulk hydrogels have been extensively studied,^113,118,120^ but not all these toughening techniques are compatible with 3D printing. Any toughening technique that requires mechanical alignment of polymer chains,^70^ freeze–thaw crystallization,^159^ or multiple solvent exchanges^160^ will affect the printed geometry and may result in distortion, deformation, or the loss of dimensional accuracy. Nonetheless, many toughening mechanisms are compatible with 3D printing, including those that increase energy dissipation through the addition of sacrificial bonds to already covalently crosslinked systems. Physical crosslinks, including ionic crosslinks,^84,158^ crystalline regimes,^80,161^ and hydrogen bonds,^162,163^ are often introduced to hydrogel systems through the incorporation of rigid nanoparticles, fibers, and small molecules within the precursor solution.^106,144–147,164^ Under tension, the weaker physical bonds break first to dissipate energy while covalent crosslinks maintain the mechanical integrity of the system. Yet, many of these physical reinforcing bonds form after 3D printing, reducing hydrogel hydration and volume, and thereby compromising the shape fidelity of such 3D printed hydrogels.^165^ The toughness of 3D printable hydrogels can also be increased by incorporating mobile moieties such as dynamic covalent bonds^166^ and slide ring crosslinks^167,168^ or by formulating them as double network hydrogels.^113,118,120^ Double network hydrogels are composed of two percolating networks – a highly crosslinked first network interpenetrated by a loosely crosslinked second network with much high polymer density.^121^ When under tension, the brittle network fractures first and dissipates energy while the second network continues to elongate under strain. The shape complexity and fidelity of double network hydrogels is limited since the 1^st^ network must be swollen in the precursor solution of the 2^nd^ network. 3D printing of double network hydrogels can be achieved if the 1^st^ network is composed of a polymer network that maintains its shape while being swollen in the 2^nd^ network precursor solution. This condition is rarely satisfied if the first network is physically crosslinked, as these networks are often too weak to maintain the printed shape during polymerization of the second covalently crosslinked network. These formulations can be 3D printed by combining precursor solutions of the 1^st^ and 2^nd^ networks to simultaneously 3D print them if each network has distinct mechanisms for crosslinking (*e.g.*, physical ionic crosslinking and covalent crosslinking).^169,170^ The shape fidelity of 3D printed double network hydrogels can be improved by pre-crosslinking precursors^171^ or through the incorporation of rheomodifying additives. However, many particles added as rheomodifiers also interact with the hydrogel matrix, thereby increasing the physical crosslink density and hence, the local stiffness of the 3D printed material.^172^

Double network hydrogels can also be formulated as granular hydrogels, resulting in double network granular hydrogels (DNGHs).^173^ DNGHs are composed of densely packed microgels that are interpenetrated and covalently crosslinked by a 2^nd^ hydrogel network. Similar to bulk double network hydrogels, DNGH toughness is primarily determined by the secondary network.^173,174^ Stress is transmitted to the microgels through the 2^nd^ network by chain entanglements and can concentrate around the poles of the microgel,^175,176^ leading to preferential chain fracture within the much stiffer microgels. Because microgels are swollen prior to their printing, DNGH systems often exhibit a higher shape fidelity than bulk double network counterparts.

**Fig. 7:**
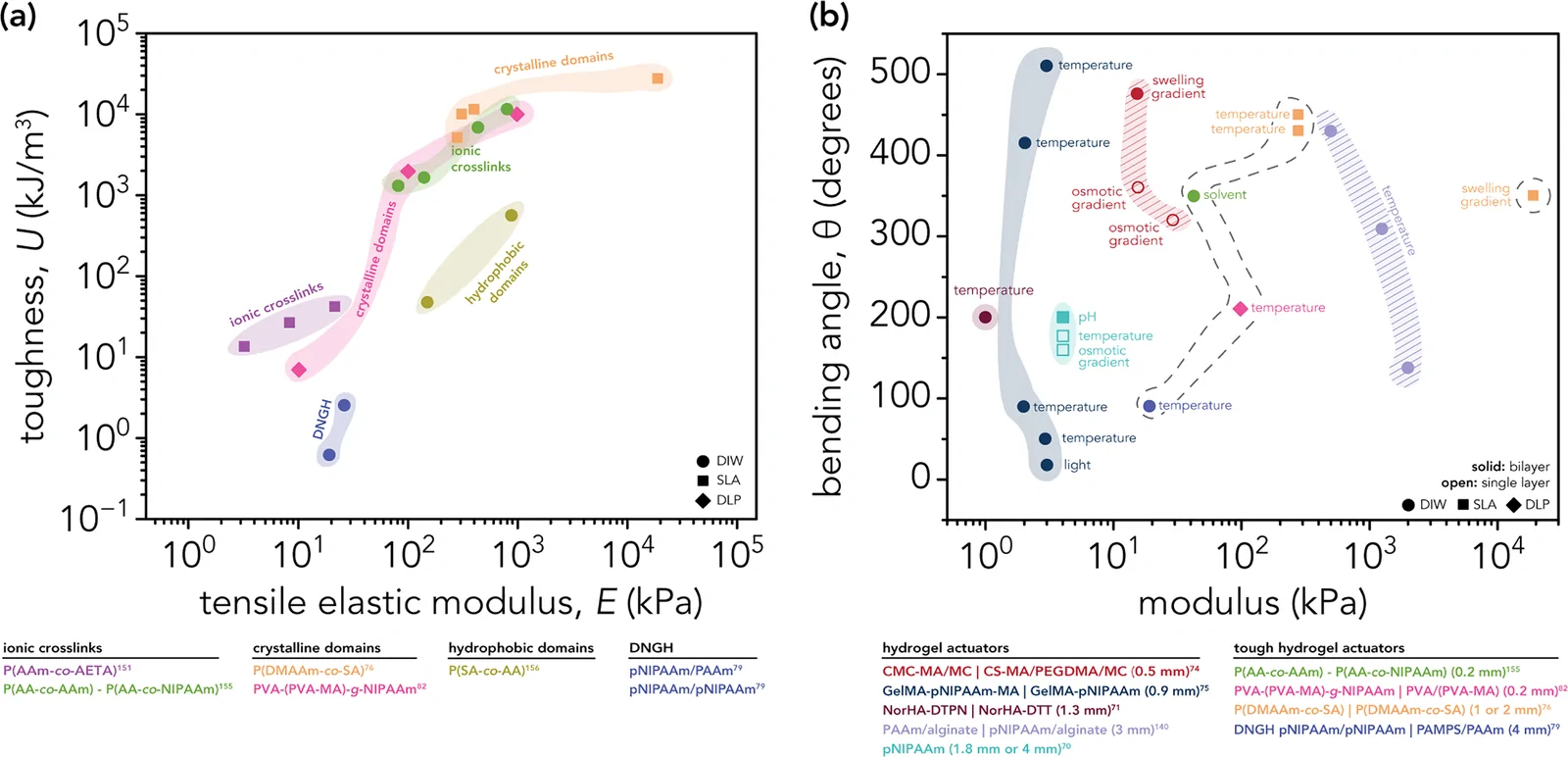
Ashby plot of (a) toughness *versus* tensile elastic modulus for 3D printed tough hydrogel actuators taken from ref. 80,83,86,154,158 and 161. The symbol shape indicates the 3D printing method used to print the actuator. Each hydrogel system is grouped by color with the toughening mechanism labeled. As elastic modulus increases, toughness generally increases. (b) Ashby plot comparing bending angle *versus* modulus for 3D printed hydrogel actuators taken from ref. 74,75,78–80,83,86,143 and 158 with the actuation mechanism labeled. The symbol indicates the 3D printing method, with a majority of the actuators being DIW-printed. The open symbols represent single layer actuators while the filled symbols represent bilayer actuators. The colors represent the different hydrogel systems with bilayer thickness in parentheses. The shading of the colored envelopes around the data points represents the modulus measurement. Filled in envelopes indicate storage modulus, the striped envelopes represent compressive modulus, while the dashed gray outline represent tensile elastic modulus and also highlight the tough hydrogel actuators. There is no correlation between bending angle and modulus, indicating that tough hydrogels can also achieve high bending angles and actuation. Most systems quantified bending angle utilized Methods 1–3.^74,75,78–80,83,86,143,158^ However, Method 4 was used for the DNGH pNIPAAm system (blue circle)^83^ and Method 5 was used for the PVA-(PVA-MA)-*g*-NIPAAm system (pink diamond),^86^ making it difficult to make a direct comparison with the rest of the data. Abbreviations: PAAm – poly(acrylamide), PAMPS – poly(2-acrylamido-2-methylpropanesulfonic acid), pNIPAAm – poly(*N*-isopropyl acrylamide), pNIPAAm-MA – poly(*N*-isopropyl acrylamide)-methacrylate, MC – methyl cellulose, alginate – sodium alginate, CS-MA – chitosan-methacrylate, CMC-MA – carboxymethyl cellulose-methacrylate, PEGDMA – poly(ethylene glycol dimethacrylate), GelMA – gelatin methacrylyol, P(SA-*co*-AA) – poly(stearyl acrylate-*co*-acrylic acid), P(AA-*co*-AAm) – poly(acrylic acid-*co*-acrylamide), P(AA-*co*-NIPAAm) – poly(acrylic acid-*co-N*-isopropylacrylamide), P(DMAAm-*co*-SA) – poly(*N,N*-dimethyl acrylamide-*co*-stearyl acrylate), P(AAm-*co*-AETA) – poly(acrylamide-*co*-[2-(acryloyloxy)ethyl]trimethylammonium chloride), PVA-(PVA-MA)-*g*-NIPAAm – poly(vinyl alcohol)-(poly(vinyl alcohol)-methacrylate)-*grafted-N*-isopropyl acrylamide), NorHA – norbornene-modified hyaluronic acid, DTPN – dithiol poly(*N*-isopropylacrylamide) crosslinker, DTT – dithiothreitol crosslinker.

Unfortunately, an increase in toughness often compromises the actuation of hydrogels because it is typically accompanied by an increase in stiffness, as summarized in Fig. 7a, resulting in higher forces required for deformation. Because actuation, quantified here by the bending angle, depends on multiple parameters, including hydrogel composition, swelling and stiffness mismatches between the active and passive layers, no clear correlation between bending angle and hydrogel modulus is evident, as demonstrated in Fig. 7b. The same trend is observed for the cast bilayer systems summarized in Fig. 2c, indicating that actuation is driven primarily by material composition rather than processing.

### 3.2. Double network granular hydrogel (DNGH)

**Fig. 8:**
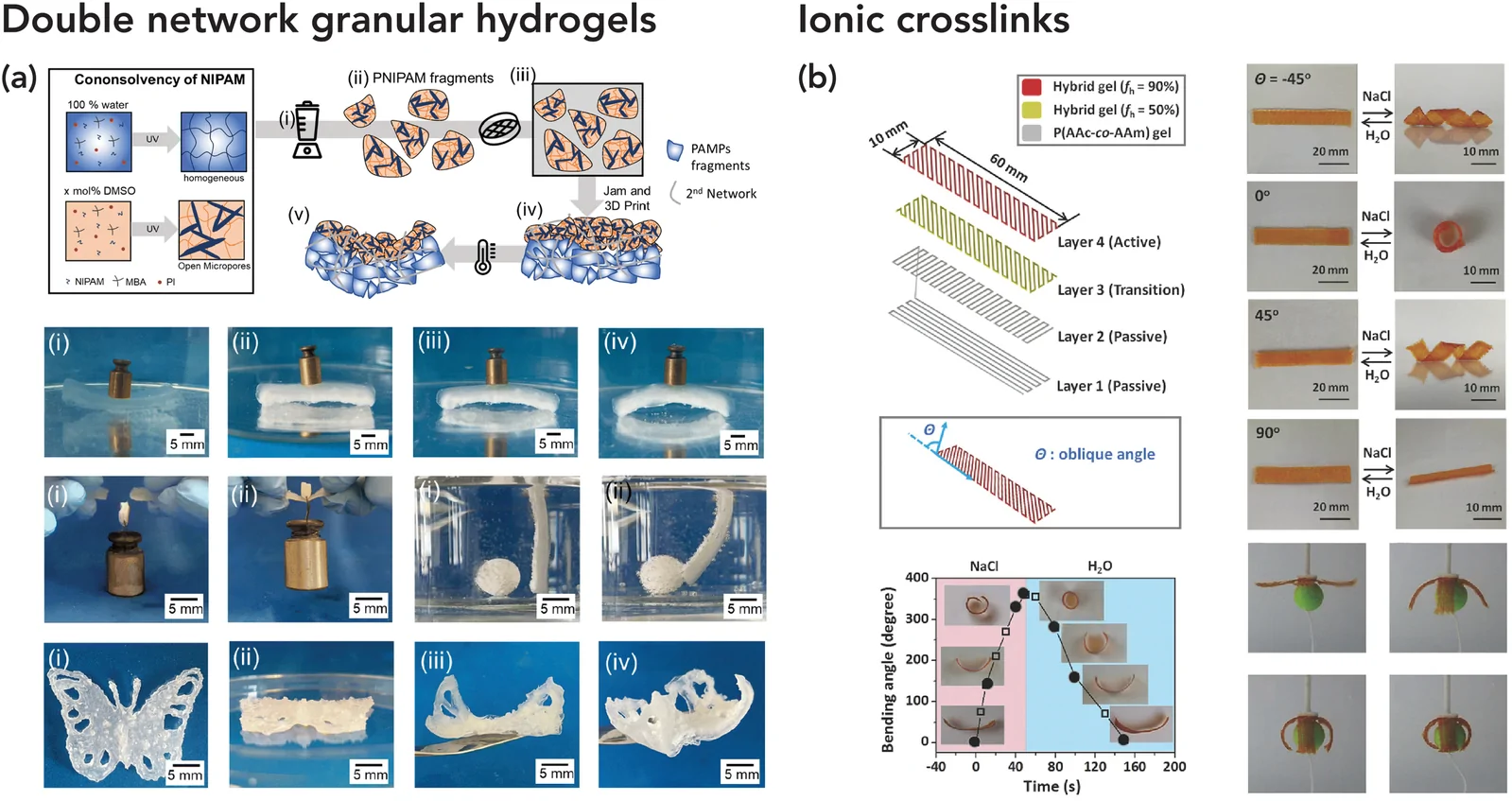
DIW-printed tough hydrogel actuators. (a) Thermoresponsive DNGH bilayer actuator composed of a pNIPAAm and PAMPS layer. Adapted and used with permission of Royal Society of Chemistry from ref. 83; permission conveyed through Copyright Clearance Center, Inc. (b) Tough hydrogel actuators composed of poly(acrylic acid-*co*-acrylamide) and poly(acrylic acid-*co-N*-isopropylacrylamide) fibers. DIW was utilized to 3D print structures that twist and bend as well as structures that can grip objects. Adapted with permission from ref. 158. Copyright 2018 John Wiley & Sons.

The thermoresponsive pNIPAAm is well-known and widely used for actuation due to its thermoresponsiveness at physiological temperatures. However, it has poor mechanical properties such that its use in actuation is limited.^44^ The mechanical properties of pNIPAAm were improved by formulating it as a DNGH composed of pNIPAAm microfragments that were interconnected by a 2^nd^ pNIPAAm or poly(acrylamide) (PAAm) network.^83^ Toughness of the thermoresponsive DNGH increased 6 times from ∼0.5 kJ m^−3^ to ∼3 kJ m^−3^ when the 2^nd^ network composed of pNIPAAm was replaced with PAAm (blue circles in Fig. 7a). However, the increase in toughness came at the expense of its thermoresponsiveness, with the PAAm network limiting the deswelling capabilities of the system. Toughness and thermoresponsiveness were combined to some extent by DIW-printing bilayers, where the active layer was composed of pNIPAAm microgels with a pNIPAAm network and the passive layer was made of poly(2-acrylamido-2-methylpropane sulfonic acid) (PAMPS) microgels connected with a PAAm network. Due to the temperature-responsive behavior of pNIPAAm and the inert behavior of PAMPS, the actuator bent 90° within 10 min and lifted a 2 g weight, corresponding to ∼20 mN of lifting force (Fig. 8a). While this result was an improvement on the mechanical properties of pNIPAAm, other toughening mechanisms were able to achieve greater mechanical properties.

### 3.3. Ionic crosslinks

An efficient way to toughen hydrogels that is also frequently used in nature is ionic reinforcement. To achieve this toughening effect, hydrogels must contain functional groups, such as carboxylic acid, that can ionize or carry a permanent charge. This feature was demonstrated by ionically crosslinking poly(acrylamide-*co*-[2-(acryloyloxy)ethyl]trimethylammonium chloride (P(AAm-*co*-AETA)) with negatively charged sulfonate-modified silica (SiO_2_) nanoparticles. Toughness and modulus increased with increasing amounts of SiO_2_ (purple squares in Fig. 7a).^154^ Similarly, metallic ions can be utilized for reinforcement. Acrylamide and *N*-isopropylacrylamide (NIPAAm) were copolymerized with acrylic acid (AA), which was ionically crosslinked with Fe^3+^ through its carboxylic groups to form poly(acrylic acid-*co*-acrylamide) (P(AA-*co*-AAm) and poly(acrylic acid-*co-N*-isopropylacrylamide) (P(AA-*co*-NIPAAm)).^158^ Ionically reinforced P(AA-*co*-AAm) was 10 times stiffer (∼800 kPa compared to 80 kPa) and approximately 9 times tougher (12 MJ m^−3^*versus* 1.3 MJ m^−3^) than P(AA-*co*-NIPAAm) (green circles in Fig. 7a). By varying the ratio of P(AA-*co*-AAm) and P(AA-*co*-NIPAAm) within the system, the toughness and elastic modulus could be tuned. Bilayers composed of P(AA-*co*-AAm) and P(AA-*co*-NIPAAm) at varying ratios were DIW-printed, and the deswelling differences between the layers upon submersion in an aqueous solution containing NaCl was leveraged to achieve actuation. The P(AA-*co*-NIPAAm) layer shrunk by 29% when immersed in solution while the P(AA-*co*-AAm) layer was unaffected. By tuning the printing angle, the actuation mode could transition from bending to twisting. When printed into a four-arm multi-layer gripper, it bent 350° within 1 min and picked up a 1.3 g ball, corresponding to a gripping force of ∼13 mN, as demonstrated in Fig. 8b.

### 3.4. Hydrophobic and crystalline domains

**Fig. 9:**
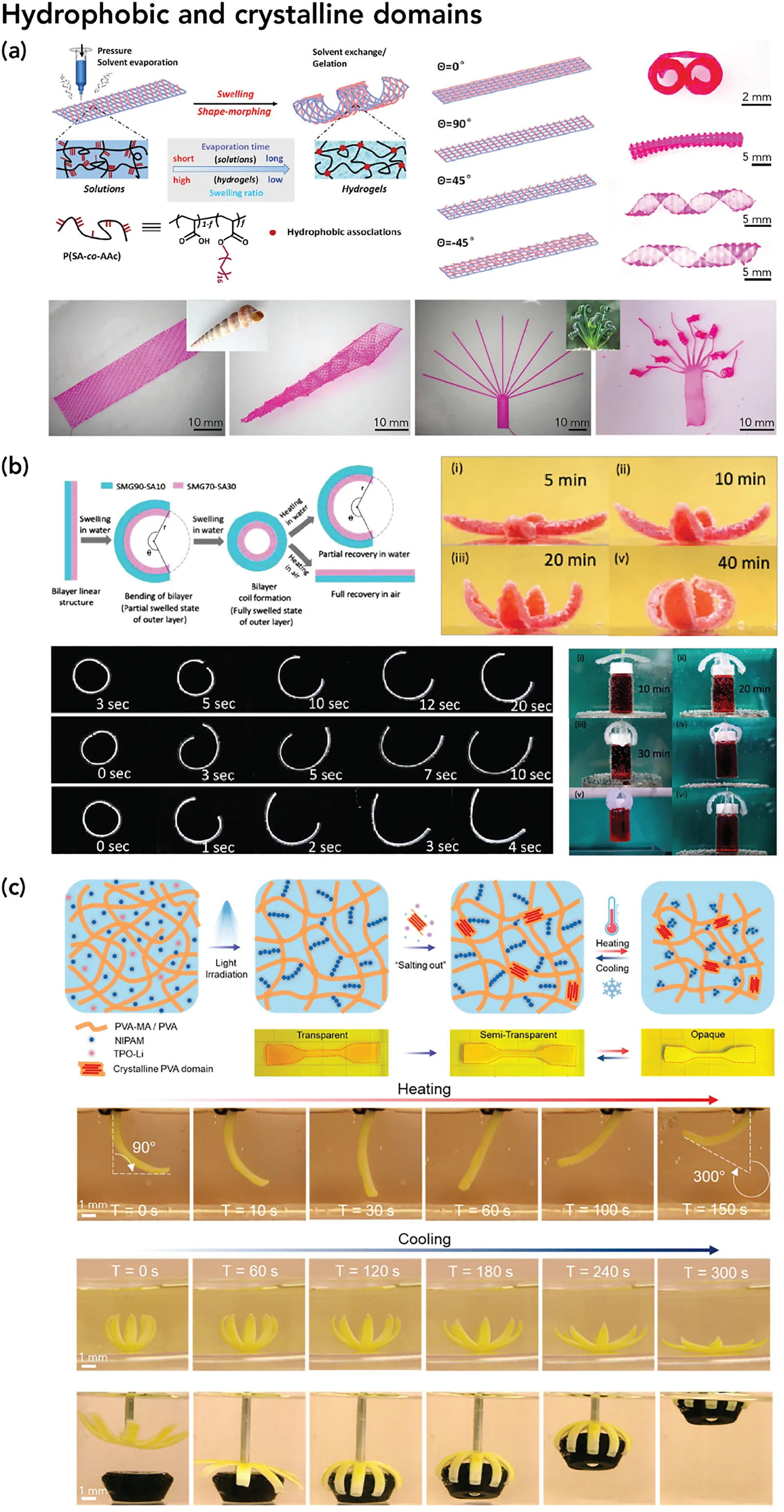
(a) Poly(stearyl acrylate-*co*-acrylic acid) (P(SA-*co*-AA)) was DIW-printed to form actuators that bend and twist depending on the print angle and evaporation time. By controlling printing angle, complex shapes found in nature can be produced. Scale bars: 2, 5, and 10 mm. Adapted with permission from ref. 161 Copyright 2021 John Wiley & Sons. (b) Bilayers of shape memory hydrogels composed of poly(*N,N*-dimethyl acrylamide-*co*-stearyl acrylate) (P(DMAAm-*co*-SA)) were printed *via* stereolithography. By controlling the amount of SA in the system, the swelling and temperature-dependent behavior of the system could be tuned. Adapted with permission from ref. 80. Copyright 2019 John Wiley & Sons. (c) DLP-printed tough actuator composed of poly(vinyl alcohol-methacrylate) (PVA-MA) grafted with pNIPAAm, which acts as thermoresponsive crosslinks. By tuning the amount of PVA-MA, PVA, and Na_2_SO_4_, the mechanical properties could be tuned. This system was tough and exhibited large actuation stresses while also achieving fast actuation speeds. Scale bars: 1 mm. Adapted with permission from ref. 86. Copyright 2021 American Chemical Society.

Tough hydrogel actuators can also be formulated in the absence of ions through the incorporation of hydrophobic, crystalline domains. This was exemplified with poly(stearyl acrylate-*co*-acrylic acid) (P(SA-*co*-AA)) that was DIW-printed into tough hydrogel actuators.^157^ The alkyl chains of the stearyl acrylate (SA) form hydrophobic domains that partially crystallize upon exposure to water, and the strong intermolecular interactions within these domains toughen the gel. The swelling and mechanical properties of the printed filaments were controlled by tuning the evaporation time of ethanol before exposure to water. The toughness of these gels increased from ∼50 to 560 kJ m^−3^ when the evaporation time increased from 0 to 1 h whereas the modulus increased from 150 to ∼900 kPa (tan circles in Fig. 7a). The elastic moduli of these gels were similar to those of gels crosslinked with Fe^3+^, but their toughness was an order of magnitude lower. Greater toughness values were reached with SA when copolymerized with *N,N*-dimethyl acrylamide (DMAAm) to form poly(*N,N*-dimethyl acrylamide-*co*-stearyl acrylate) (P(DMAAm-*co*-SA)).^80^ The crystallization of the SA was utilized to control the thermoresponsive behavior of the hydrogel, as the crystalline regions would melt at elevated temperatures, decreasing the elastic modulus and increasing the water content of the hydrogel. By tuning the temperature and concentration of SA within the copolymer, a large range of elastic moduli (∼280 kPa to ∼20 MPa) and toughness values (∼5.1 MJ m^−3^ to ∼28 MJ m^−3^) were achieved (orange squares in Fig. 7a). Actuation was demonstrated by SLA printing a 1 mm thick bilayer where each layer was composed of P(DMAAm-*co*-SA) with varying amounts of SA. At room temperature, a swelling gradient between layers drove actuation, and the system achieved 350° within 2.25 h. At 60 °C, the same system reached 450° within 1 min. When the thickness was doubled to 2 mm, the bending angle slightly decreased to 430° and actuation time increased to 5 min (orange squares in Fig. 7b). The crystalline domains also imparted shape memory behavior to the material, which was utilized for actuation, as demonstrated in Fig. 9b. When SLA-printed into a multi-arm gripper and immersed in 50 °C water, the gripper bent and picked up a 40 g vial (∼400 mN of force). Once the temperature decreased below the glass transition temperature of the system, the deformation was locked in place as the crystalline regimes formed again, allowing the vial to be transported. This process was reversed by increasing the temperature.

Crystalline domains can also be attained with poly(vinyl alcohol) (PVA) and its derivatives, which aggregate and form nanocrystalline domains when exposed to salt such as Na_2_SO_4_. However, PVA is nonresponsive on its own. Stimuli-responsive behavior was imparted to PVA by grafting pNIPAAm to methacrylated poly(vinyl alcohol) (PVA-MA) to form PVA-(PVA-MA)-*g*-pNIPAAm.^86^ The amount of crystallization was controlled by adjusting the ratio of pristine PVA with PVA-MA as well as the salt concentration. The elastic modulus varied by two orders of magnitude (10 kPa to 1 MPa) while the toughness was tuned three orders of magnitude (∼10 kJ m^−3^ to 10 MJ m^−3^) by increasing the molar concentration of Na_2_SO_4_ from 0.1 M to 1 M (pink diamonds in Fig. 7a). This extensive range of mechanical properties demonstrates the versatility of crystallization as a toughening mechanism. When DLP-printed into a bilayer composed of a passive PVA/PVA-MA layer and an active PVA/(PVA-MA)-*g*-pNIPAAm layer, it acted as a thermoresponsive gripper. Upon exposure to elevated temperatures, the bilayer bent approximately 210° within 150 s and was able to lift a 1 g weight without deforming the gripper (∼10 mN gripping force), as shown in Fig. 9c.

There is no universally optimal toughening strategy for hydrogel actuators as the elastic modulus and toughness are system dependent. The best approach depends on the material's chemistry, such as ionic crosslinking for polyelectrolyte systems or domain formation in crystallizable or partially hydrophobic systems. For all of these 3D printed tough hydrogel actuators, the tensile elastic modulus positively correlated with the toughness. This is a significant improvement over typical 3D printed actuators, whose tensile mechanical properties are often not even reported or are so low that shear modulus or compressive modulus are measured instead.

### 3.5. Toughness and actuation

The toughness of reinforced hydrogel actuators positively correlates with their stiffness, as demonstrated in Fig. 7a. Remarkably, the toughness, and hence stiffness, of these actuators does not show a clear correlation with their bending angles, as demonstrated in Fig. 7b, where the bending angle of toughened hydrogels can range from 17.5 to 510° with actuation times varying from 0.5 to 135 min.^74,75,78–80,83,86,143,158^ This comparison suggests that tough hydrogel actuators can achieve similarly large bending angles as those achieved by untoughened actuators, despite their significant improvement in mechanical properties.

### 3.6. 3D printing of tough hydrogels

Many studies fabricate tough actuators as unstructured bilayer structures, limiting the range of achievable actuation modes to bending. Leveraging 3D printing can overcome this constraint by enabling spatially programmable structures and locally varying compositions. For example, poly(stearyl acrylate-*co*-acrylic acid) (P(SA-*co*-AA)) was direct ink written into a bilayer actuator containing an orthogonal grid-like pattern. The direction of curvature was controlled by changing the filament printing angle, as shown in Fig. 9a. Similarly, vat photopolymerization enabled the fabrication of more complex bilayer structures, such as flowers or multi-arm grippers, as demonstrated in Fig. 9b and c.

3D printing also offers control over actuation time. For example, if tough hydrogels are printed into latticed and grid-like structures, their response time decreases as water diffusion throughout the system is accelerated,^79,86^ albeit often at the expense of mechanical properties. Overall, these examples demonstrate that 3D printing not only expands the accessible actuation modes of tough hydrogel actuators but can also be leveraged to program their geometry, composition, and dynamic response.

## 4. Outlook for the development of 3D printable tough hydrogel actuators

Many currently available hydrogel-based actuators are optimized for actuation, at the expense of their mechanical properties such that they often exhibit limited toughness and fatigue resistance. The toughness of hydrogel actuators can be increased by introducing reversible bonds or crystalline domains. However, these toughening mechanisms often result in stress–strain hysteresis^177–179^ and damage accumulation within the material which limits the lifetime of actuators subjected to cyclic loading. Although some studies have explored the development of tough hydrogels with minimized hysteresis,^180^ their application in actuators remains limited. This limitation was partially addressed in double network granular hydrogel systems, which exhibit low hysteresis and fast shape recovery.^181^ If the interstitial spaces are functionalized with conductive materials, such as poly(3,4-ethylenedioxythiophene) poly(styrene sulfonate) (PEDOT:PSS), they could be used as piezoresistive strain sensors and, if further functionalized with ions, as Joule heaters. Indeed, a DNGH-based Joule heater was integrated into a thermoresponsive DNGH layer that was combined with a passive piezoresistive DNGH layer to actuate the bilayer structure through closed-loop control. The piezoresistive DNGH was able to sense the degree of deformation of the actuator and determine if actuation should be maintained through Joule heating or not.^182^ The low stress–strain hysteresis resulted in minimal signal drift.^181^ However, connecting stimuli-responsive microfragments with a nonresponsive 2^nd^ network improves the mechanical properties of the responsive hydrogel at the expense of its actuation. This example illustrates the difficulty of encoding actuation within these systems without the use of an external power source.

Many hydrogel actuators either suffer from poor fatigue resistance or lack fatigue resistance characterization all together. Indeed, few studies demonstrate repeatable actuation beyond 10 cycles, and even fewer conduct long-term fatigue testing. This shortcoming may arise from the challenges associated with fatigue characterization in hydrogels, particularly because drying can occur during testing and influence the measured response. Drying can be significantly decelerated by replacing water with less volatile solvents, such as deep eutectic solvents^183^ or ionic liquids.^159,184^ However, solvent exchange can alter the viscoelastic behavior and mechanical properties of these hydrogels. While there have been studies on fatigue-resistant hydrogels,^185^ these approaches have yet to be applied in hydrogel actuators. For comparison, a human heart valve beats ∼3 billion times over the course of a lifetime.^186^ Synthetic materials often cannot achieve these cyclic loads that nature can due to their inability to self-heal. Self-healing can be imparted to synthetic hydrogels, for example by adding motives that form hydrogen^181,187^ and ionic bonds^87^ or using thiol–ene click chemistry.^188^ Alternatively, unpolymerized monomers can be embedded within the material where mechanical force triggers polymerization.^189,190^ Thixotropic gels can also be used as a self-healable sacrificial network.^122^ For actuators to be viable in everyday applications, they would benefit from these self-healing abilities to prolong their lifetime.

Currently, hydrogel actuators are optimized for one specific application, whether that is bending a specified amount, producing a high output force, or carrying a load. This review focused on the bending capabilities of hydrogel actuators as this is the predominant metric utilized in soft gripper development. However, the practical applications of these untethered hydrogel actuators beyond grasping an object immersed in water and transporting it are currently limited. The mechanical performance and fatigue resistance are still lacking, along with slow actuation times. For many of these untethered systems whose actuation relies on water diffusion, speed will always be a limiting factor unless the system is engineered in a way to achieve stimuli-responsive bistability due to an anisotropic dimensional change. For example, a PDMS system with embedded glass fibers was direct ink written.^191^ By utilizing geometry, beam buckling theory, and anisotropic swelling, bistability within the system was triggered upon swelling in organic solvent. The rapid collapse of the system allowed it to act like an “on/off” switch. This could be utilized for microfluidic valves or flow regulators, where a rapid switch may be needed. However, while bistable systems have been demonstrated for elastomers,^12^ there are fewer studies on this mechanism for hydrogels.^58^ Indeed, another limitation for hydrogel actuators is their need to be constantly hydrated. This constraint lends them well for the development of biomedical applications such as organ replacements and artificial muscles.

Rendering 3D printable tough hydrogels biocompatible represents yet an even greater challenge. Many hydrogels optimized for mechanical properties or actuation are not biocompatible. Indeed, biocompatible hydrogels tend to be either stiff and brittle, such as poly(ethylene glycol) (PEG), or stretchable and soft, such as alginate and gelatin. Fewer biocompatible hydrogels are stimuli-responsive, which is crucial for actuation. Chitosan and alginate are promising candidates due to their pH-responsiveness and ionic crosslinking, respectively, although their mechanical properties are far weaker than synthetic systems. Recently, a tough PVA-based heart was direct ink written.^43^ In this case, actuation was driven by an external pneumatic system, further demonstrating the current limitations of untethered actuators. Similarly, polymer-based artificial muscles typically utilize external actuation sources such as electricity to achieve maximum displacement and force in a short amount of time.^65^ For hydrogel-based artificial muscles, there is still a wide gap in performance. Most hydrogel actuators cannot achieve the same actuation stresses demonstrated by mammalian muscles which is of order 100s of kPa, whereas that of hydrogel is of order 1 kPa, as summarized in Table 1 and Fig. 3. Similarly, the work density, which is the amount of work delivered per unit volume, for muscles is ∼10 kJ m^−3^ whereas it is three orders of magnitude smaller for hydrogel artificial muscles at 10^−2^ kJ m^−3^.^65^ Recently, a poly(acrylamide-*co*-acrylic acid) (P(AAm-*co*-AA)) hydrogel system was able to demonstrate a work density of 15 kJ m^−3^ by deforming the system and then using ionic crosslinking to store the elastic potential energy.^70^ When the ionic crosslinks were released upon light irradiation, the stored energy was quickly released as the system contracted, mimicking the quick energy release demonstrated in mammalian muscles. While this is an impressive performance for a hydrogel-based system, an external source was still required to achieve the initial deformation.

Despite the significant interest in untethered hydrogel actuators, their current applications remain constrained by their low fatigue resistance, self-healing capabilities, biocompatibility, and actuation speed. Additionally, the advantages offered by 3D printing have not yet been fully realized. Although 3D printing enables precise spatial patterning and complex geometries, future progress is needed to develop effective strategies that leverage these capabilities for enhanced functionality, performance, and control.

## 5. Conclusion

The rapid advancement of 3D printable tough hydrogel actuators has opened up new possibilities in material design and functionality. However, the field currently lacks a standardized framework for defining, characterizing, and quantifying key properties such as toughness and actuation performance. Establishing consistent metrics would allow different material systems to be fairly compared and evaluated. This review indicates that currently reported tough 3D printed hydrogel actuators can achieve similar actuation to standard 3D printed hydrogel actuators while achieving better mechanical properties. This advancement marks a significant step towards enabling the practical use of untethered hydrogel-based soft robots and actuators. Yet, many applications would require stiffer, stronger or more fatigue resistant hydrogels. We envisage that advancements in mechanical properties, combined with the processability of precursors through additive manufacturing, will open up new avenues for their use, for example, in soft robotics and biomedicine.

## Author contributions

Allison L. Chau: conceptualization, data curation, writing – original draft. Esther Amstad: conceptualization, writing – review and editing, supervision, project administration

## Conflicts of interest

There are no conflicts to declare.

## Data Availability

No primary research results, software or code have been included and no new data were generated or analysed as part of this review.
